# Absent right and persistent left superior vena cava: troubleshooting during a challenging pacemaker implant: a case report

**DOI:** 10.1186/1756-0500-7-462

**Published:** 2014-07-21

**Authors:** Jacques Rizkallah, John Burgess, Vikas Kuriachan

**Affiliations:** 1 Libin Cardiovascular Institute of Alberta, University of Calgary, C829 Foothills Medical Centre, 1403 29 Street NW, Calgary, AB T2N 2 T9, Canada

**Keywords:** Persistent left superior vena cava, Absent right superior vena cava, Challenging pacemaker implant

## Abstract

**Background:**

Venous anomalies of the thorax can occur in isolation or in association with complex congenital heart disease. The incidence of an absent right superior vena cava in the setting of a persistent left superior vena cava is very rare in the general population with only a dozen cases documented in the medical literature. Such venous anomalies can make for very challenging electronic cardiac device implantation. We report our challenging dual chamber pacemaker implant in a patient with such complex anatomy and focus on our implantation technique that helped achieve adequate lead positioning.

**Case presentation:**

A 73-year-old Caucasian female with degenerative complete heart block presented for dual chamber permanent pacemaker implant. Lead implantation was very challenging due to abnormal and rare vena cava anatomy; a persistent left superior vena cava drained directly into the coronary sinus and the right brachiocephalic vein drained directly into the left persistent superior vena cava as the patient had an absent right superior vena cava . Adequate right ventricular lead positioning was achieved following numerous lead-stylet manipulations and careful looping in the atria to redirect its trajectory to the ventricular apex.

**Conclusion:**

Abnormal superior vena cava development is uncommon and can lead to technical challenges when venous access is required during various interventional procedures. Pre-operative imaging can help identify such challenging anatomy allowing appropriate operative planning; careful patient selection is warranted for venography given the risk of contrast nephrotoxicity.

## Background

Venous anomalies of the thorax can occur in isolation or in association with complex congenital heart disease. The incidence of an absent right superior vena cava (SVC) in the setting of a persistent left SVC is very rare in the general population with only a dozen cases documented in the medical literature. We report our challenging dual chamber pacemaker implant in a patient with such complex anatomy and focus on our implantation technique that helped achieve adequate lead positioning. We also review the medical literature on this topic.

## Case presentation

**Figure 1:**
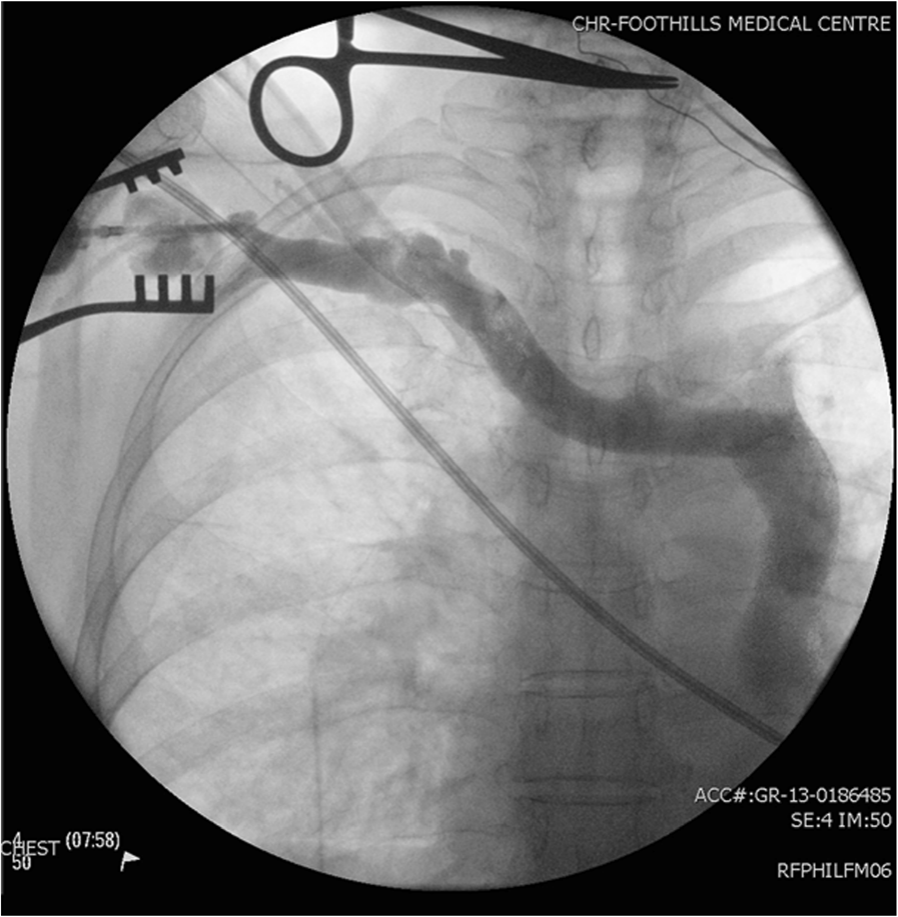
Central venogram depicting absent right and persistent left SVC.

**Figure 2:**
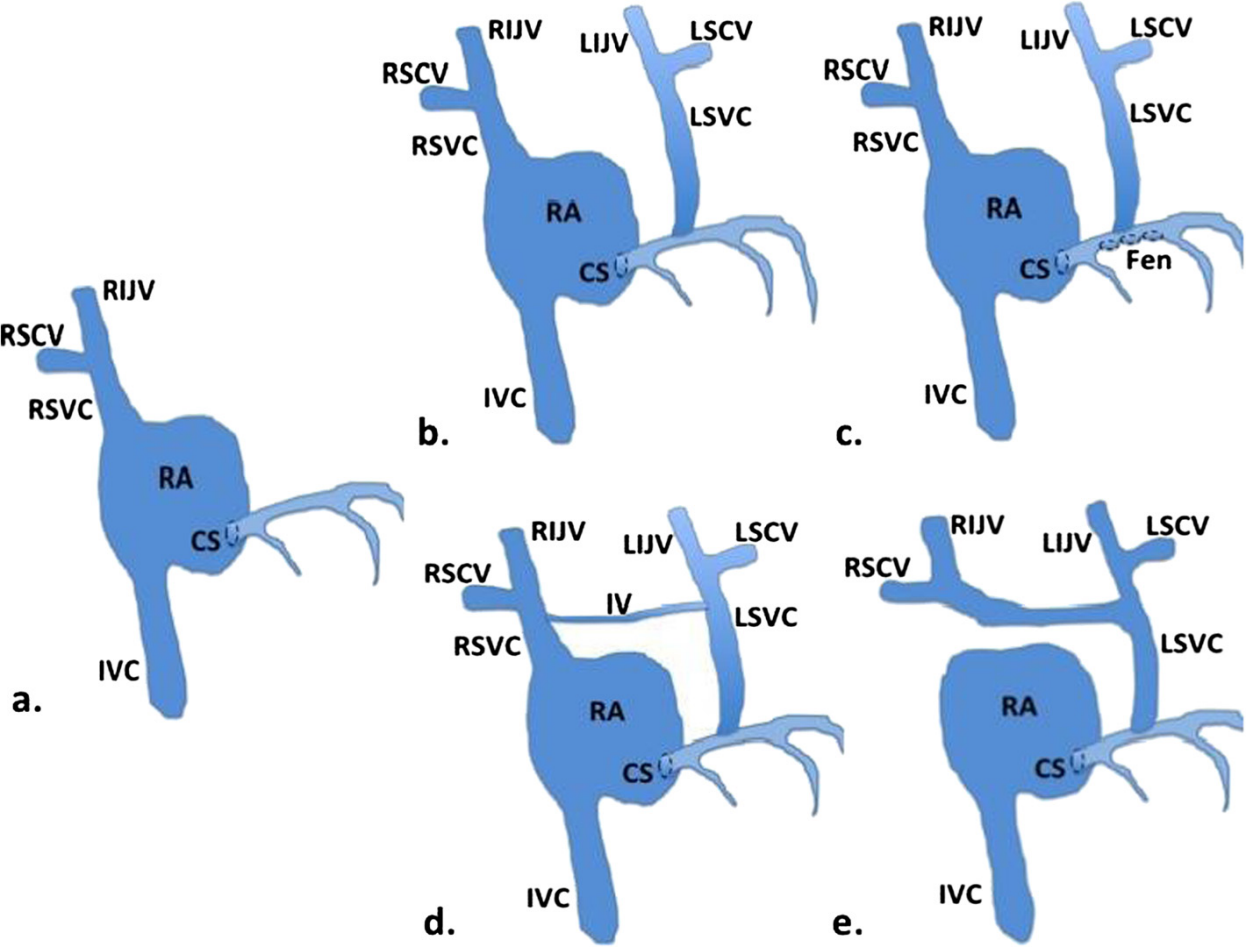
**Examples of certain persistent left superior vena cava anatomic variations. a.** Typical venous drainage into the right atrium. **b.** Persistent left superior vena cava and its tributaries draining into the coronary sinus. **c.** Persistent left superior vena cava draining into the left atrium by means of an unroofed coronary sinus. **d.** Persistent left superior vena cava draining into the coronary sinus and also connected to the right superior vena cava by an innominate vein. **e.** Persistent left superior vena cava with an absent right superior vena cava as identified in this case report. CS = Coronary sinus; Fen = Fenestrations; IV = Innominate vein; IVC = Inferior vena cava; LIJV = Left internal jugular vein; LSCV = Left subclavian vein; LSVC = Left superior vena cava; RA = Right atrium; RIJV = Right internal jugular vein; RSCV = Right subclavian vein; RSVC = Right superior vena cava.

**Figure 3:**
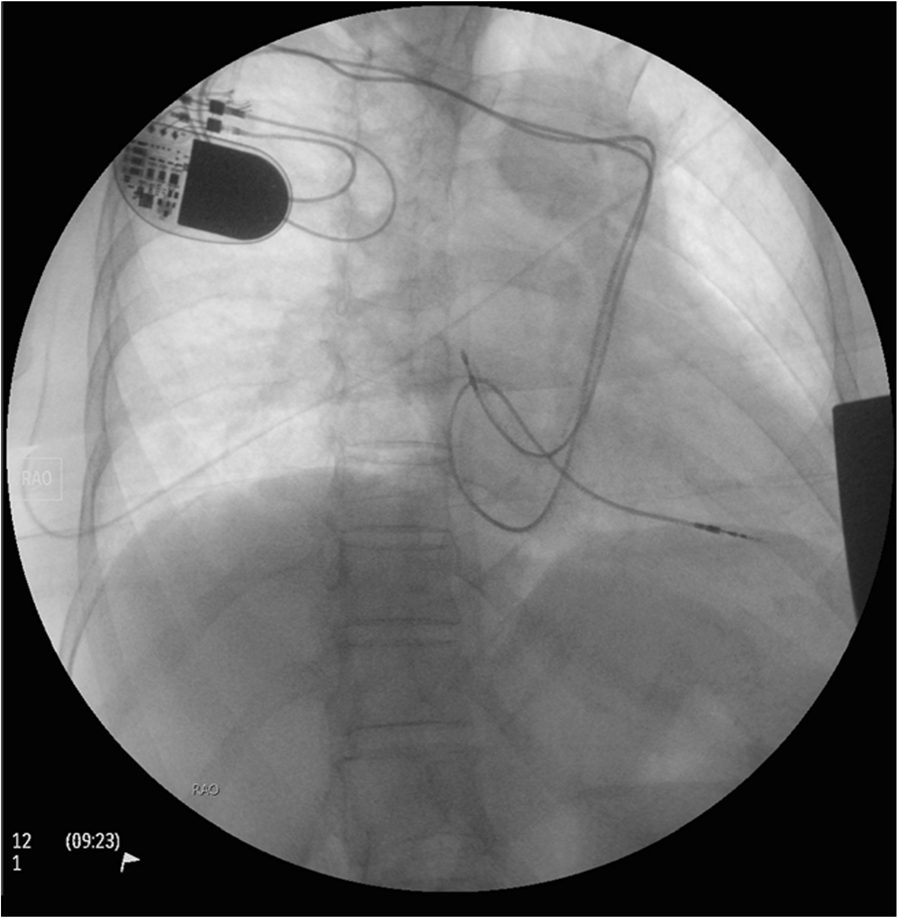
Chest X-ray depicting final position of the right atrial and ventricular pacemaker leads; note how the right ventricular lead is looped in the right atrium to redirect its trajectory towards the ventricular apex.

A previously healthy 73-year-old Caucasian female presents to the clinic with a history of progressive fatigue and dyspnea on exertion over the past couple of months. She denied angina, palpitations, syncope or any other associated symptoms and did not have any cardiovascular disease risk factors. On examination, she was stable and in no distress. Her blood pressure was 148/66 mmHg with a regular pulse of 48 beats per minute. She had no clinical evidence of heart failure on cardiovascular examination but was found to have an S4 on auscultation and cannon A waves on assessment of her jugular venous pressure (JVP). Her exam was otherwise unremarkable. Twelve-lead electrocardiogram during the clinic visit revealed complete heart block with a junctional escape rhythm at 49 beats per minute with right bundle branch block morphology; her 12-lead electrocardiogram 3 months earlier also revealed evidence of underlying conduction disease with a prolonged PR interval and right bundle branch block while in sinus rhythm. Trans-thoracic echocardiography displayed normal left ventricular size and function with mild degenerative changes of the mitral and aortic valves consistent with age related changes. There was no laboratory evidence of any metabolic or ischemic etiologies for the conduction disease. Given her age, clinical presentation, underlying conduction disease in the setting of no significant structural abnormalities on echocardiography, and lack of significant comorbidities, the patient likely had age related degenerative conduction disease. Significant coronary artery disease could not be excluded but was very unlikely given the lack of ischemic symptoms and cardiovascular disease risk factors along with normal cardiac size and function. She was arranged to have a permanent pacemaker implant for the symptomatic complete heart block and will have further risk stratification for possible coronary artery disease following the pacemaker implantation.The patient was brought to the operating theater following informed consent. Initial venous access and lead implantation was attempted across the left cephalic vein but aborted due to difficulty in delivering the pacing lead into the right ventricular (RV) cavity; the venous trajectory was that of a persistent left superior vena cava (SVC) draining directly into the coronary sinus. Right cephalic venous access was subsequently obtained and to our surprise there was an anomalous venous trajectory on the right implantation side as well (Figure 1); on intra-operative venography is was evident that the right brachiocephalic vein drained directly into the left persistent SVC as the patient had an absent right SVC (Figure 2). Steering the right atrial (RA) pacing lead into the RA cavity was not very difficult however positioning it in the right atrial appendage required careful manipulation. Once in the RA cavity, the straight stylet of the RA pacing lead was exchanged for the curved “J” stylet and clock-wise torque was applied during further advancement of the lead to direct it anteriorly in the optimal atrial appendage position (Figure 3). Positioning the RV pacing lead was a challenge since the pacing lead was gaining atrial access across the coronary sinus (CS) OS which is located very close to the RV inlet however the CS OS directs the lead away from the RV inlet towards the lateral free wall of the RA. We were able to redirect the RV pacing lead into the RV by reflecting its tip off the RA free wall thus looping the lead around into the RA and back into the RV (Figure 3); in order to reflect the tip of the RV pacing lead off the RA free wall it is very important to withdraw the straight lead stylet back a few centimeters to ensure the tip of the lead is no longer stiff and thus reduce the risk of atrial perforation. Once across the tricuspid valve, the straight stylet can be advanced further if necessary to guide the pacing lead to the RV apex. Alternative methods that could be attempted to direct the RV pacing lead into the RV cavity in such complex anomalous venous anatomy includes adding significant distal curve to the lead stylet or using one of the curved long venous access sheaths that are typically used for CS OS canulation during cardiac-resynchronization therapy device implantation.

Following pacemaker implantation, the patient was monitored overnight in the cardiac telemetry unit and discharged home the following day. Her symptoms of fatigue and dyspnea on exertion resolved following device implant.

### Discussion

In this case report, we document our pacemaker implantation technique in a patient with a very rare venous anomaly involving an absent right and persistent left superior vena cava.

Venous anomalies of the thorax can occur in isolation or association with congenital heart disease (CHD). A persistent left SVC (Figure 2) can be identified incidentally in 0.5% of the general population and 4% of patients with CHD. Typically when present the left SVC is alongside a smaller caliber right SVC and the left SVC is usually not associated with a brachiocephalic vein. In the incidental and benign setting, the left SVC drains directly into the coronary sinus; when draining directly into the left atrium, the persistent left SVC is usually associated with CHD with the most common being atrial septal defects and coarctation of the aorta [1,2].

Anomalies of the right SVC can include drainage to the left atrium, low right atrial insertion, aneurysmal dilatation, and anomalous left brachiocephalic vein drainage to the right SVC [1]. Persistent left SVC with an absent right SVC is very uncommon in the general population and occurs in 0.07 to 0.13% of patients who have CHD with viscera-atrial situs solitus [3].

Long-term prognosis in abnormal SVC development is good in the absence associated CHD [4]. Venous anomalies however can make central venous access and in particular pacemaker lead implantation very challenging; this includes single and dual pacemaker implantation along with cardiac resynchronization therapy whereby the insertion of any lead (atrial, ventricular, or coronary sinus leads) would be challenging due to the unfamiliar venous course which does not conform with the pre-formed shapes of the venous access tools utilized during these procedures. These venous anomalies can also cause procedural challenges in any other types of thoracic and cardiac surgeries especially if the physician is not familiar with the course of the venous anomaly at hand along with other associated congenital abnormalities. Pre-operative imaging with venography, magnetic resonance imaging, computed tomography, or agitated-saline contrast echocardiography can help identify cases of challenging venous anatomy allowing appropriate operative planning; careful patient selection is warranted given the risk of contrast nephrotoxicity.

The pacemaker lead implantation techniques we described above to overcome the challenges of this particular anomalous venous drainage case may not apply or be effective in all patients with similar or differing anatomy. It is therefore essential to evaluate the underlying venous anatomy at hand objectively using intra-operative venography or pre-operative imaging if possible when an anomalous anatomy is suspected. Once the anatomy is clarified, the technique that is the safest and least invasive for a particular scenario should be attempted first. Failure to implant pacing leads intravenously may warrant consideration for surgical epicardial lead implantation. In the setting of failed endocardial defibrillator lead implantation attempts, subcutaneous defibrillators can also be considered.

## Consent

Written informed consent was obtained from the patient for publication of this case report and any accompanying images. A copy of the written consent is available for review by the Editor-in-Chief of this journal.

## Conclusion

Abnormal SVC development is uncommon and can lead to technical challenges when venous access is required during various interventional procedures. Pre-operative imaging can help identify such challenging anatomy allowing appropriate operative planning; careful patient selection is warranted for venography given the risk of contrast nephrotoxicity.

## Abbreviations

CHD: Congenital heart disease; CS: Coronary sinus; JVP: Jugular venous pressure; RA: Right atrium; RV: Right ventricle; SVC: Superior vena cava.

## Competing interests

The authors declare that they have no competing interests

## Authors’ contributions

JR, JB, and VK were equally involved in the care of the patient and case report composition. JR was the artist and creator of Figure 2. All authors read and approved the final manuscript.
